# Magnetic Ligand Fishing Using Immobilized Cyclooxygenase-2 for Identification and Screening of Anticoronary Heart Disease Ligands From *Choerospondias axillaris*

**DOI:** 10.3389/fnut.2021.794193

**Published:** 2022-01-31

**Authors:** Miaomiao Chi, Hongsen Wang, Zhankuan Yan, Lei Cao, Xun Gao, Kunming Qin

**Affiliations:** ^1^Jiangsu Key Laboratory of Marine Pharmaceutical Compound Screening, Jiangsu Ocean University, Lianyungang, China; ^2^Jiangsu Original Drug Research and Development Co., Ltd., Lianyungang, China; ^3^Jiangsu Institute of Marine Resources Development, Jiangsu Ocean University, Lianyungang, China; ^4^Co-Innovation Center of Jiangsu Marine Bio-industry Technology, Jiangsu Ocean University, Lianyungang, China

**Keywords:** cyclooxygenase-2, magnetic ligand fishing, *Choerospondias axillaris*, UPLC-Q-Exactive Orbitrap-MS/MS, traditional Chinese medicine

## Abstract

Inhibition of cyclooxygenase-2 (COX-2) activity is an effective way for treatment of coronary heart disease. And as an important source of COX-2 inhibitors, bioactive compounds of *Choerospondias axillaris* and pharmacological mechanisms remained lacking in prospective researches. Therefore, for the purpose of accelerating the discovery of natural products targeting designed inhibitors, the COX-2 microreactor composed of functionalized microspheres and magnetic ligand fishing was developed and applied in *Choerospondias axillaris*, and the physicochemical properties of the COX-2 functionalized microspheres were characterized using Fourier transform infrared spectroscopy (FT-IR), vibrating sample magnetometer (VSM), scanning electron microscopy (SEM), and transmission electron microscopy (TEM). Furthermore, the bioactive compounds singled out from ethanol decoction without prepurification were dissociated and identified by ultraperformance liquid chromatography plus Q-Exactive Orbitrap tandem mass spectrometry (UPLC-Q-Exactive Orbitrap-MS/MS). Consequently, 21 bioactive compounds consisting of 6 organic acids, 8 flavonoids, and 7 others were separated and characterized from *Choerospondias axillaris*, which were reported to participate in the COX-2 inhibitory pathway to varying degrees. Therefore, this method could provide a prospective solution for the extraction and identification of active pharmaceutical ingredients and the rapid screening of some enzyme inhibitors in the complex mixtures.

## Introduction

In most countries, the prevalence of the cardiovascular disease is still on the rise. *Choerospondias axillaris* has been used in China to treat the coronary heart disease as early as the eighth century (1). Modern pharmacological researches also showed that *Choerospondias axillaris* was used to treat cardiovascular diseases because of its antiarrhythmia and antimyocardial ischemia effects (2, 3). However, due to its complex chemical composition, there are few studies on the specific functional components representing the specific pharmacological activities of *Choerospondias axillaris* and their corresponding targets (4).

The results of network pharmacology and pharmacokinetic studies suggest that the flavonoids and organic acids may be the material basis for the prevention and treatment of coronary heart disease in *Choerospondias axillaris* (5). Flavonoids may mediate the cGMP-PKG signaling pathway, and organic acids such as gallic acid, protocatechuic acid, and chlorogenic acid may mediate the Wnt signaling pathway to jointly play the role of preventing and treating coronary heart disease. Moreover, the prevention and treatment of coronary heart disease may be related to related biological processes such as stress response, immune-inflammatory response, signal transduction, and atherosclerotic plaque instability by *Choerospondias axillaris*. COX-2 was reported to be a key enzyme that participated in platelet aggregation, and regulate the resistance of peripheral blood vessels and the distribution of renal blood flow (6–8). Therefore, it is of great importance that started with COX-2 to study the active pharmaceutical compounds and their mechanism of action from *Choerospondias axillaris*.

Screening methods for active compounds of COX-2 inhibitors in Chinese medicine mainly include classic separation and activity testing methods (9), chip technology (10), high-throughput screening methods (11), and virtual ligand screening methods using structural information (12); however, the above methods have disadvantages such as large workload, long cycle, and easy loss of active compounds. Since drugs in the body exert their pharmacological effects by interacting with a variety of related biological target molecules, such as enzymes, receptors, DNA, and RNA, during the development of the disease, the ligand capture technology has become an efficient screening method guided by biological activity and based on affinity screening, which can not only quickly and target the active ingredients of traditional Chinese medicines, but also help to explain the mechanism of action of traditional Chinese medicines at the molecular level, thereby providing new methods and ideas for the treatment of diseases.

Magnetic ligand fishing is a combination of specific ligand affinity and magnetic separation, which has been proven to be a convenient and effective tool for extracting biologically active ingredients in extracts of plant. Though modern analytical techniques such as HPLC, LC-MS, and SPE-HPLC-NMR can achieve rapid identification and full structural characterization of constituents in complex matrixes, it was difficult that provide related information about biologically active ingredients. Magnetic ligand fishing detects active ingredients by binding affinity directly from the crude extract without the process of prepurification (13). However, when some ingredients produced false-positive results in ligand fishing due to the non-specific binding of immobilized enzymes, the risk can be effectively reduced by docking the protein structure of COX-2. When combined with proteins, graphene oxide (GO) can maintain the ligand structure and physiological activity because of the hydrophobic and hydrogen bonding with proteins, which indicated that GO can be used in magnetic ligand fishing. Magnetic ligand fishing has been applied to isolate the ligands of human serum albumin (14), α-amylase (15), α-glucosides (16), cyclooxygenase-1 (COX-1) (17), and acetylcholine ester (18).

With the continued need for fast and sensitive methods for the identification of COX-2 inhibitory compounds of natural origin, we established the first application of *Choerospondias axillaris* by a combination of GO-based magnetic ligand fishing and UPLC-Q-Exactive Orbitrap-MS/MS. Because of the binding affinity of the COX-2 ligand, the use of the newly magnetic nanomaterials in ligand fishing is not limited to the crude extracts of *Choerospondias axillaris*, and the materials showed excellent specificity, good precision, and high reusability in the experiment.

## Materials and Methods

### Chemicals and Reagents

Graphene oxide was purchased from the Nanjing Jicang Nano Technology Co., Ltd (Nanjing, China). Human recombinant COX-2 and methanol (HPLC grade) were supplied by Sigma (St. Louis, MO, USA). 3-aminopropyltriethoxysilane (APTES), tetraethyl orthosilicate (TEOS), glutaraldehyde (25% (w/v) aqueous solution), acetic acid (HPLC grade), ethanol, acetonitrile, FeCl_3_·6H_2_O (99%), FeCl_2_·4H_2_O (99%) were obtained from Aladdin (Shanghai, China). Water used throughout the work was produced by a Milli-Q ultrapure water system (Millipore, Bedford, Massachusetts, USA).

### Instrumental

The magnetite nanoparticles were characterized by transmission electron microscopy (TEM), Fourier-transform infrared (FT-IR) spectroscopy, scanning electron microscope (SEM), and vibration sample magnetometer (VSM). TEM micrographs were obtained by the JEM2100F system (JEOL, Japan). SEM microstructure of nanoparticles was recorded by the S4800 system (Hitachi, Japan). The FT-IR spectra were recorded using KBr pellets on Nicolet iS50 spectrophotometer (Thermo Fisher Scientific, Waltham, Massachusetts, USA). The magnetic properties of nanoparticles were investigated by a VSM7407 (Lakeshore, Louisiana, USA).

### High-Performance Liquid Chromatography Analysis

Chromatographic separation was performed on an analytical symmetry C18 column (250 × 4.6 mm i.d., 5 μm, Waters, Milford, Massachusetts, USA). The isocratic mobile phase consisted of water-methanol (15:85, v/v) with a flow rate of 1.0 ml/min was prepared for celecoxib analysis at 25°C and the chromatograms were acquired at 254 nm.

### UPLC-Q-Exactive Orbitrap-MS/MS

The UPLC-MS/MS analysis of the eluent was performed on an analytical Shim-pack XR-ODS II (75 × 3.0 mm i.d., 1.7 μm, Shimadzu, Kyoto). The UPLC system mainly consisted of an Ultimate 3000 series ultra-high-performance liquid chromatography (Thermo Fisher Scientific, San Jose, USA) equipped with an online degasser, a quaternary pump, an autosampler, and a column temperature compartment; it was connected to a Q-Exactive Orbitrap tandem mass spectrometer (Thermo Fisher Scientific, San Jose, USA) *via* an electrospray ionization interface (ESI). The mobile phase consisted of C (0.4% acetic acid in water) and D (acetonitrile) at 30°C, and the elution gradient as shown in Table 1. All the mobile phases were prepared daily.

**Table 1:** The elution gradient of UPLC-Q-Exactive Orbitrap-MS/MS.

**Time (min)**	**Flow rate (ml/min)**	**A (%)**	**B (%)**
0–2	0.3	95–85	5–15
2–3	0.3	85–75	15–25
3–7	0.3	75–55	25–45
7–9	0.3	55–40	45–60
9–10	0.3	40–25	60–75
10.01–12	0.3	95	5

Mass spectra were acquired in both the positive and negative ion mode through full MS and higher-energy collisional dissociation data-dependent MS/MS analysis. Full-scan high-resolution accurate-mass data acquisition captures all the sample data, enabling identification of untargeted compounds and retrospective data analysis without rerunning the samples.

Ion source parameters were shown as follows: spray voltage −3.0 kV (negative polarity) and 3.3 kV (positive polarity), sheath gas 35 arbitrary units, auxiliary gas 10 arbitrary units, capillary temperature 350°C, S-lens RF level 55, auxiliary gas heater temperature 350°C. Automatic gain control was set at 1e6, and the maximum injection time at 50 ms, and the isolation window at 2.0 *m/z*. The collision energy was varied in the range of 20–40 eV and scan range *m/z* 60–900 to obtain representative product ion spectra. The mass tolerance window was set to 5 ppm for the two analysis modes (2002/657/EC). Data analysis and processing have been performed using the Xcalibur 4.1 software (Thermo Scientific, USA).

### Preparation of GO@Fe_3_O_4_@SiO_2_-COX-2 Nanoparticles

Based on the previous report, some modifications were made to coat COX-2 on the GO layer (19).

The magnetic GO@Fe_3_O_4_ (MGO) nanoparticles were prepared by the chemical coprecipitation of Fe^3+^ and Fe^2+^. At first, 0.50 g of GO was dispersed in 100 ml of distilled water and heated to 70°C. A total of 2.16 g of FeCl_3_·6H_2_O and 0.80 g of FeCl_2_·4H_2_O were dissolved in 40.0 ml of distilled water, and then added to the aforementioned dispersion suspension. After adjusting the pH to 10 with ammonia water (25%, w/v), the dispersion solution continued to be 70°C for 6 h. The whole process of the reaction was carried out under a stream of pure nitrogen. When the reaction finished, the suspension was filtered and washed three times with distilled water and ethanol, respectively.

Second, 6.3 ml of distilled water and 2.1 ml of TEOS were stirred for 3 min and sonicated for 1 min to obtain a milky solution. The milky solution was added to 49.5 ml of absolute ethanol including 0.50 g of magnetic GO@Fe_3_O_4_ nanoparticles. After stirring at 0°C for 10 min, 2.0 ml of glutaraldehyde was added dropwise to the dispersion solution. Then, the reaction lasted for 10 h. At last, separated by a magnet, GO@Fe_3_O_4_@SiO_2_ (SMGO) nanoparticles were sequentially washed with 2% HNO_3_, distilled water, and absolute ethanol in turn.

Finally, 0.10 g of SMGO nanoparticles were suspended in 50 ml ethanol-water solution (1:1, v/v), and adjusted the pH to 3–4 with 0.1 mol L^−1^ HCl. Followed by 1.0 ml APTES dropwise, the mixture was continued to be stirred for 5 h at 50°C. SMGO-NH_2_ nanoparticles were separated by a permanent magnet and washed 4 times with ethanol. After being suspended in 5.0 ml of 5% glutaraldehyde solution for 1 h, the SMGO-NH_2_ nanoparticles were retrieved by magnetic separation and washed with Tris-HCl buffer (100 mM, pH 8.0) three times to remove excess glutaraldehyde. Then shaken SMGO-NH_2_ nanoparticles with 2.0 ml of tris-HCl buffer containing 50 units of COX-2 at 37°C for 2 h, the COX-2 functionalized magnetic microspheres (SMGO-COX-2) were synthesized.

### Magnetic Ligand Fishing in *Choerospondias axillaris*

A total of 5.0 g of *Choerospondias axillaris* powder was extracted with 500 ml of 75% (v/v) ethanol three times in parallel at 85°C for 1.5 h. After filtering on three layers of gauze of the diameter of 4.5 mm, the extraction solutions were combined, freeze-dried, and dissolved to 2 mg ml^−1^. A total of 5 mg of COX-2 functional magnetic microspheres were suspended in 2 ml of extraction solution and incubated at 37°C for 30 min. Then, it was washed 3 times with 2 ml Tris-HCl buffer (pH 8.0) to remove non-specific binding ligands, and incubated with 1 ml of methanol for 1 h to dissociate the extracted ligand. After the nanoparticles were separated by a magnet, the elution solution had been filtered through a 0.22 μm membrane and then introduced into UPLC-Q-Exactive Orbitrap-MS/MS for analysis. Meanwhile, celecoxib was incubated with MGO, SMGO, and SMGO-inactive COX-2.

## Results and Discussion

### Characterization of the Nanoparticles

**Figure 1:**
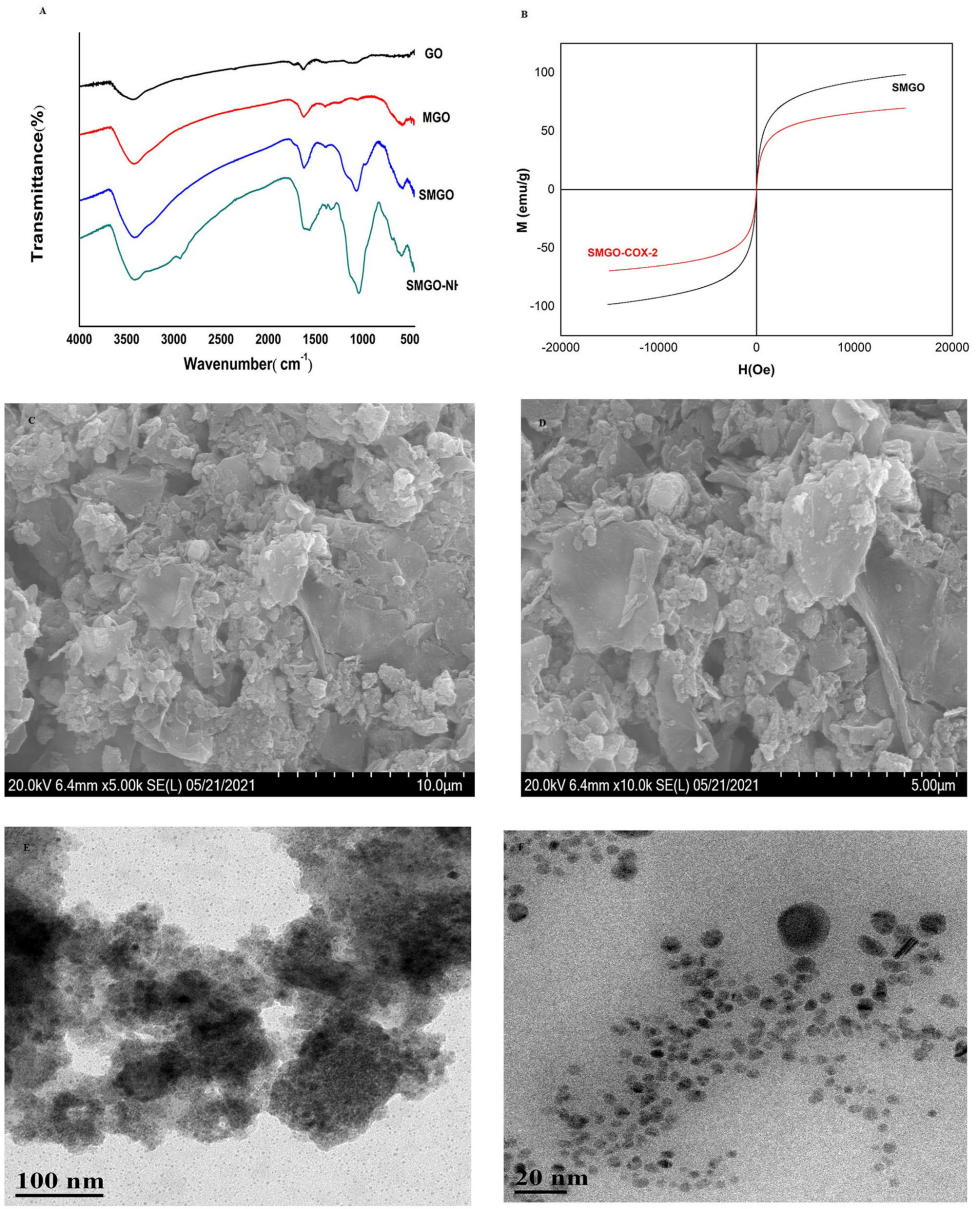
**(A)** Fourier-transform infrared (FT-IR) spectra of GO, MGO, SMGO, and SMGO-NH2. **(B)** Magnetization curves of SMGO, SMGO@COX-2. **(C,D)** SEM images of SMGO@COX-2; **(E,F)** TEM images of SMGO@COX-2.

Fourier-transform infrared spectroscopy analysis was used to record the surface functional groups and chemical bonds of synthetic materials. The infrared spectra of GO, MGO, SMGO, SMGO-NH_2_ were shown in Figure 1A. The peak at 541 cm^−1^ was due to the tensile vibration of Fe-O-Fe. The strong absorption band at 1,066 cm^−1^ was attributable to the symmetric and asymmetric vibrations of Si-O-Si, indicating that GO was covered by Fe_3_O_4_ and SiO_2._ The absorption at 1,300 cm^−1^ was attributed to the stretching vibration of C-N, indicating that SMGO was modified with the amino groups. Therefore, the results of FTIR analysis confirmed the formation of SMGO-NH_2_ nanoparticles. The magnetic properties of the material were studied by VSM at room temperature. According to the curve in Figure 1B, the saturation magnetization of SMGO and SMGO-COX-2 was 98.42 and 69.60 emu·g^−1^, respectively, which were enough to separate nanomaterials from the sample solution under an external magnetic field. Compared with SMGO, the magnetization of SMGO-COX-2 was reduced by 28.82 emu·g^−1^, which was attributed to the enzyme grafted on the surface of SiO_2_. The morphology study of SMGO@COX-2 nanocomposite was shown in Figures 1C,D. Previous studies showed that GO was a single flake layer (19). After combining Fe_3_O_4_ particles, the surface becomes rough with spherical or elliptical substances. After coating SiO_2_, the agglomeration phenomenon was reduced. After being connected to COX-2, the flake layer of GO was almost invisible, and the gaps created by the prevention of agglomeration of SiO_2_ were also filled, and the entire surface became rougher and fuller. The structural morphology of SMGO@COX-2 nanocomposite was shown in Figures 1E,F. The black dots (SMGO@COX-2) on the cross-section were evenly anchored on the surface of the nanomaterial, and the shell-like layered structure could be seen inside the material.

### Assay Verification

**Figure 2:**
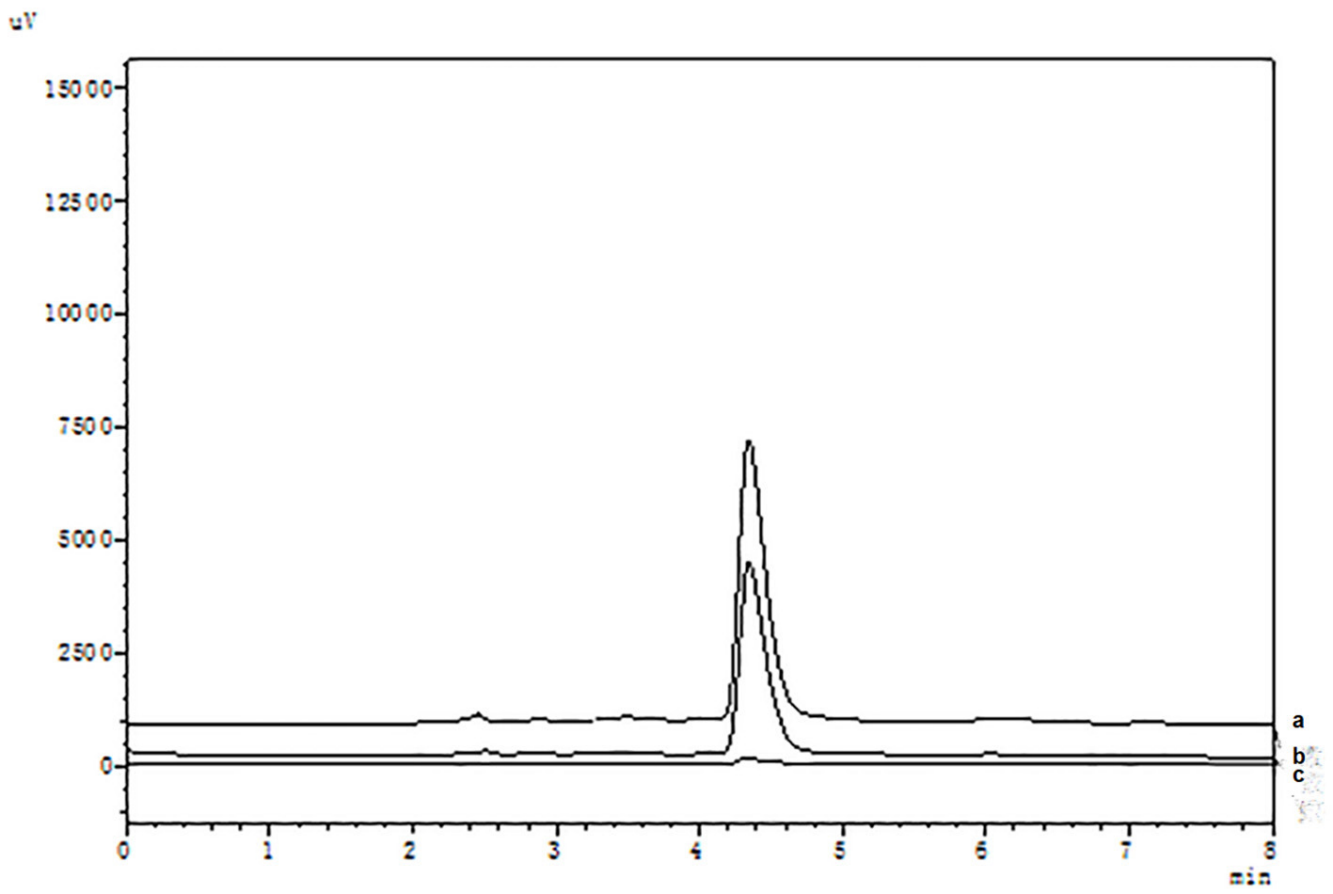
High-performance liquid chromatography (HPLC) chromatograms of celecoxib (a) and ligand fishing assay eluent by SMGO-COX-2 (b), and SMGO (c).

It is well known that compared with COX-1, celecoxib exhibits an extremely high-selective inhibitory effect on COX-2. The HPLC analysis were shown in Figure 2. When the COX-2 was bound to the surface of SMGO, the peak area of celecoxib in the elution solution increased significantly. Meanwhile, as shown in Supplementary Figure S1, when the inactive COX-2 was bound to the surface of SMGO, the peak area of celecoxib in the elution solution could be ignored. And extracted with MGO and SMGO, the celecoxib in the elution solution was rarely detected. Therefore, the results demonstrated the specificity of this magnetic ligand fishing for the screening of COX-2 ligands.

The stable activity of the immobilized enzyme COX-2 was a key prerequisite for capturing the same number of ligands during a series of binding-dissociation cycles. Therefore, the repeatability of SMGO@COX-2 was evaluated by specifically capturing celecoxib. To avoid the loss of SMGO@COX-2 nanoparticles during the cycles, it was necessary to expose them to a magnetic field for 1 min. After 5 consecutive association-dissociation cycles, as shown in Supplementary Figure S2, the binding efficiency was still as high as 90% of the first cycle by calculating the peak area of celecoxib in the elution solution. The results showed a stable combination of SMGO and COX-2 and a high COX-2 ligand-binding capacity. After 5 days of storage at 4°C, as shown in Supplementary Figure S3, there was no significant loss on the celecoxib binding efficiency (RSD <8%, *n* = 5), and three batches of SMGO@COX-2 showed similar binding efficiency (RSD <10%).

### Identification of COX-2 Inhibitors by UPLC-Q-Exactive Orbitrap-MS/MS

**Figure 3:**
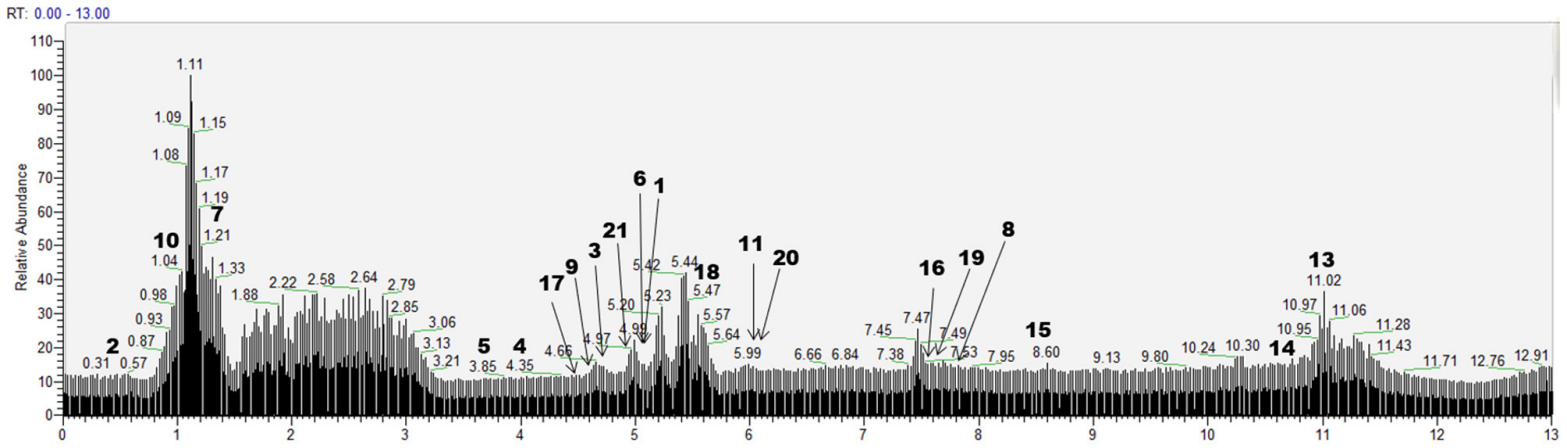
Base peak chromatogram of the elution solution of *Choerospondias axillaris* by UPLC-Q-Exactive Orbitrap-MS/MS in the negative mode.

**Figure 4:**
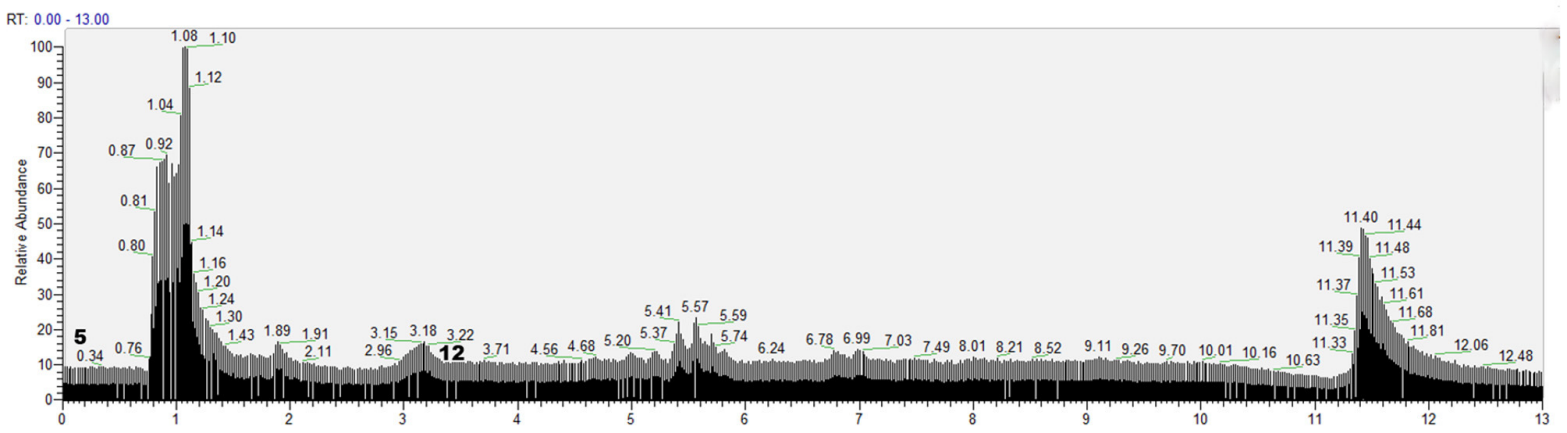
Base peak chromatogram of the elution solution of *Choerospondias axillaris* by UPLC-Q-Exactive Orbitrap-MS/MS in the positive mode.

According to the retention time of each component, mass spectrum information, and related references, 21 COX-2 ligands were preliminarily determined in the eluted solution using magnetic ligand fishing technique as listed in Table 2 and Figures 3, 4. Both negative and positive ions were applied in the UPLC-Q-Exactive Orbitrap-MS/MS system to analyze the ligands of COX-2 separated from *Choerospondias axillaris*. The results in Figure 3 showed that the eluted solution had better resolution of the references and more observable fragment ions in negative ion mode, which mainly attributed to the large amounts of phenolic acids and flavonoids.

**Table 2:** Identification of elution solution by UPLC-Q-Exactive Orbitrap-MS/MS.

**No**.	**Compounds**	**Chemical formula**	**R_**t**_ (min)**	**Ion mode**	**Observe mass (Da)**	**Error (ppm)**	**MS/MS**	**Confidential levels**
1	Hydroquinone	C_6_H_6_O_2_	5.03	–	109.02915	−3.325	81.03429[M-CO-H]^−^	2
2	Succinic acid	C_4_H_6_O_4_	0.43	–	117.01905	−2.409	73.02924[M-CO_2_-H]^−^	2
3	Protocatechualdehyde	C_7_H_6_O_3_	4.69	–	137.02397	−3.265	93.03431[M-CO_2_-H]^−^	2
4	Protocatechuic acid	C_7_H_6_O_4_	3.99	–	153.01891	−2.758	109.02916[M-CO_2_-H]^−^	2
							81.03448[M-CO_2_-CO-H]^−^	
5	Isovanillin	C_8_H_8_O_3_	0.18	+	153.05443	−1.246	/	2
			3.80	–	151.03976	−2.035	136.01616[M-CH_3_-H]^−^	
							108.02154[M-CH_3_-CO-H]^−^	
6	Vanillic acid	C_8_H_8_O_4_	5.02	–	167.03418	−4.801	152.01100[M-CH_3_-H]^−^	1
							108.02141[M-CH_3_-CO_2_-H]^−^	
7	Gallic acid	C_7_H_6_O_5_	1.2	–	169.01341	−4.95	125.02403[M-CO_2_-H]^−^	2
8	Caffeic acid	C_9_H_8_O_4_	7.77	–	179.03459	−2.19	135.04480[M-CO_2_-H]^−^	1
9	Syringaldehyde	C_9_H_10_O_4_	4.63	–	181.05003	−3.325	166.02614[M-CH_3_-H]^−^ 151.00313[M-2CH_3_-H]^−^	2
10	Quinic acid	C_7_H_12_O_6_	1.05	–	191.05539	−3.776	173.04510[M-H_2_O-H]^−^	2
11	Citric acid	C_6_H_8_O_7_	6.03	–	191.01825	−7.726	111.00851[M-2H_2_O-CO_2_-H]^−^	1
							87.00853[M-2CO_2_-OH]^−^	
12	Pantothenic acid	C_9_H_17_NO_5_	3.39	+	220.11786	−0.405	202.10732[M-H_2_O+H] ^+^	2
							124.07572[M-C_2_H_8_O_4_+H] ^+^	
13	Palmitic acid	C_16_H_32_O_2_	11.04	–	255.23247	−1.874	/	3
14	Pinocembrin	C_15_H_12_O_4_	10.72	–	255.06573	−2.615	213.05482[M-C_2_H_2_O-H]^−^	2
							151.00323[M-C_8_H_8_-H]^−^	
15	Naringenin	C_15_H_12_O_5_	8.56	–	271.06122	0.086	177.01860[M-C_6_H_7_O-H]^−^	2
							151.00316[M-C_6_H_7_O-2CH_3_-H]^−^	
16	Kaempferol	C_15_H_10_O_6_	7.57	–	285.04062	0.557	257.04523[M-CO-H]^−^	2
17	Catechin	C_15_H_14_O_6_	4.56	–	289.07175	0.039	245.08098[M-CO_2_-H]^−^	1
							203.07045[M-C_4_H_6_O_2_-H]^−^	
18	Ellagic acid	C_14_H_6_O_8_	5.45	–	300.99771	−4.254	283.99567[M-H_2_O-H]^−^	1
							273.04050[M-CO-H]^−^	
							229.04938[M-CO-CO_2_-H]^−^	
19	Quercetin	C_15_H_10_O_7_	7.65	–	301.03439	−3.275	151.00316[M-C_7_H_6_O_2_-CO-H]^−^	1
							178.99800[M-C_7_H_6_O_2_-H]^−^	
							107.01353[M-C_8_H_6_O_3_-CO_2_-H]^−^	
							121.02917[M-C_8_H_4_O_5_-H]^−^	
20	Taxifolin	C_15_H_12_O_7_	6.05	–	303.04874	−7.543	285.04025[M-H_2_O-H]^−^	1
							125.02387[M-C_9_H_6_O_4_-H]^−^	
21	Rutin	C_27_H_30_O_16_	4.96	–	609.14532	−1.293	300.02667[M-C_12_H_20_O_9_-H]^−^	1
							301.03378[M-C_12_H_21_O_9_-H]^−^	
							271.02411[M-C_12_H_20_O_9_-H_2_O-H]^−^	
							255.02911[M-C_12_H_22_O_11_-H]^−^	

*Confidential level 1: Compounds that matched to reference standard*.

*Confidential level 2: Compounds that matched to robust spectral or literatures*.

*Confidential level 3: Compounds that classified*.

#### Identification of Flavonoids

A total of 8 flavonoids have been identified in the elution solution of *Choerospondias axillaris*, accounting for more than 38%. In the negative mode ESI-MS spectra, the [M-H]^−^ ion was observed for all the compounds. In the negative mode ESI-MS^2^ spectra, the [M-A-H]^−^ ion was observed for 6 compounds, such as compounds 14, 15, 16, 19, 20, and 21 (A represents various groups with more than 6 carbon atoms). The [M-CH_3_-H]^−^ ion was observed for 2 compounds, such as compounds 9 and 15. The [M-H_2_O-H]^−^ ion was observed for 2 compounds, such as compounds 20 and 21. The [M-CO-H]^−^ ion was observed for 2 compounds, such as compounds 16 and 19. The [M-CO_2_-H]^−^ ion was observed for 2 compounds, compounds 17 and 19. From this result, it can be seen that [M-H]^−^ is the most fragmented ions, followed by [M-A-H]^−^.

#### Identification of Phenolic Acids

A total of 6 phenolic acids have been identified in the elution solution of *Choerospondias axillaris*, accounting for more than 28%. In the negative mode ESI-MS spectra, the [M-H]^−^ ion was observed for all the compounds. In the negative mode ESI-MS^2^ spectra, the [M-CO_2_-H]^−^ ion was observed for all the compounds. The [M-CH_3_-H]^−^ ion was observed for 1 compound, compounds 6. The [M- H_2_O-H]^−^ ion was observed for 1 compound, compounds 11. The [M-CO-H]^−^ ion was observed for 1 compound, compounds 4. From this result, it can be seen that [M-H]^−^ and [M-CO_2_-H]^−^ are the most fragmented ions in phenolic acids.

#### Identification of Other Compounds

A total of 6 other compounds including 2 aromatic aldehydes, 2 organic acids, and 2 phenols, have been identified in the elution solution of *Choerospondias axillaris*, accounting for more than 28%. In the negative mode ESI-MS spectra, the [M-H]^−^ ion was observed for all the compounds. In the negative mode ESI-MS^2^ spectra, the [M-CO-H]^−^ ion was observed for 2 phenols and 1 aromatic aldehyde, compounds 1, 5, and 18. The [M-CO_2_-H]^−^ ion was observed for 1 phenol and 1 aromatic aldehyde, compounds 3 and 18. The [M- H_2_O-H]^−^ ion was observed for 1 organic acid and 1 aromatic aldehyde, compounds 10 and 18. And in the positive mode ESI-MS spectra, the [M+H]^−^ ion was observed for all the compounds. In the positive mode ESI-MS spectra, the [M- H_2_O +H]^−^ ion was observed for 1 compound, compounds 12. From this result, it can be seen that [M-H]^−^ and [M-CO-H]^−^ are the most fragmented ions in other compounds.

### Evaluate of COX-2 Inhibitors in *Choerospondias axillaris*

Consistent with our results, 21 bioactive compounds were reported to participate in the COX-2 inhibitory pathway in different forms. It is well known that LPS-induced reactive oxygen species production leads to COX-2 induction, and compounds 1, 3, 9, 15, 16, 19, and 20 were found to effectively inhibit the production of LPS-induced COX-2 metabolites (20–23). It has been reported that compound 8 suppressed UVB-induced COX-2 expression by blocking Fyn kinase activity (24). Meanwhile, compounds 4, 6, 13, and 14 suppressed the NF-κB signaling pathways to limit the expression of COX-2 (25–28). Differently, compounds 7, 10, 17, 18, and 21 were proven to reduce COX-2 levels by reducing nitric oxide overproduction from inducible nitric oxide synthase (29–31). Considering the aforementioned facts, we can still conclude that, the compounds analyzed by UPLC-Q-Exactive Orbitrap MS/MS were consistent.

## Conclusion

*Choerospondias axillaris* has complex components and diverse structures, which has the effect on multiple targets. Network pharmacology showed that their anticoronary heart disease action pathway was mainly through anti-inflammation, which provided theoretical support to choose the target of COX-2. And this study aimed to establish a fast and simple method to target and screen out the active compounds that interacted with COX-2 from *Choerospondias axillaris*, and it was the first time that synthesized COX-2 magnetic microspheres were applied to extract COX-2 inhibitors. Meanwhile, the COX-2 functionalized magnetic microspheres in this method had excellent specificity, good precision, low cost, and high-throughput screening efficiency. Therefore, the method could be used to immobilize macromolecular targets and provide convenient conditions for screening many specific biologically active compounds from complex natural products.

## Data Availability Statement

The original contributions presented in the study are included in the article/Supplementary Material, further inquiries can be directed to the corresponding author/s.

## Author Contributions

MC helped in writing the original draft, data curation, and formal analysis. HW was involved in methodology, project administration, and validation. ZY helped with the investigation and resources. LC worked with software and visualization. XG was involved in the reviewing and editing and funding acquisition. KQ helped with supervision and conceptualization. All authors contributed to the article and approved the submitted version.

## Funding

This work was supported by the National Natural Science Foundation of China (No. 82104349) and Project Funded by the Priority Academic Program Development of Jiangsu Higher Education Institutions (PAPD).

## Conflict of Interest

Authors HW and ZY were employed by Jiangsu Original Drug Research and Development Co., Ltd. The remaining authors declare that the research was conducted in the absence of any commercial or financial relationships that could be construed as a potential conflict of interest.

## Publisher's Note

All claims expressed in this article are solely those of the authors and do not necessarily represent those of their affiliated organizations, or those of the publisher, the editors and the reviewers. Any product that may be evaluated in this article, or claim that may be made by its manufacturer, is not guaranteed or endorsed by the publisher.

## Supplementary Material

Supplementary Figure S1 The HPLC chromatograms of celecoxib (a) and ligand fishing assay eluent by SMGO-COX-2 (b), MGO (c), SMGO-inactive COX-2 (d), and SMGO (e).

Supplementary Figure S2 The HPLC chromatograms of celecoxib in five consecutive association-dissociation cycles of ligand fishing assay eluent by SMGO-COX-2.

Supplementary Figure S3 The HPLC chromatograms of celecoxib in ligand fishing assay eluent by SMGO-COX-2 during five days of storage at 4°C.
