# Supplementary figures and images for: Magnetic Ligand Fishing Using Immobilized Cyclooxygenase-2 for Identification and Screening of Anticoronary Heart Disease Ligands From Choerospondias axillaris
Source: Front Nutr. 2022 Jan 31;8:794193. doi: 10.3389/fnut.2021.794193 (PMC8841743; doi:10.3389/fnut.2021.794193)

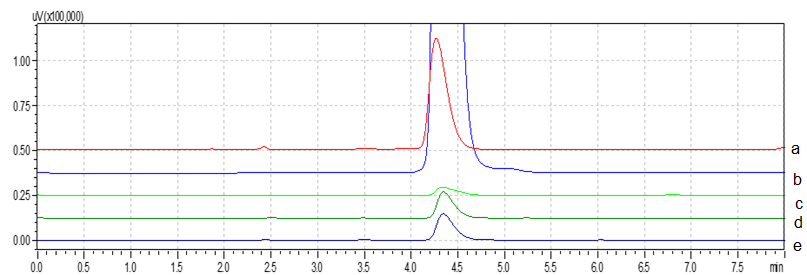

Supplement: Supplementary Figure S1 — The HPLC chromatograms of celecoxib (a) and ligand fishing assay eluent by SMGO-COX-2 (b), MGO (c), SMGO-inactive COX-2 (d), and SMGO (e). [file Image_1.PNG]

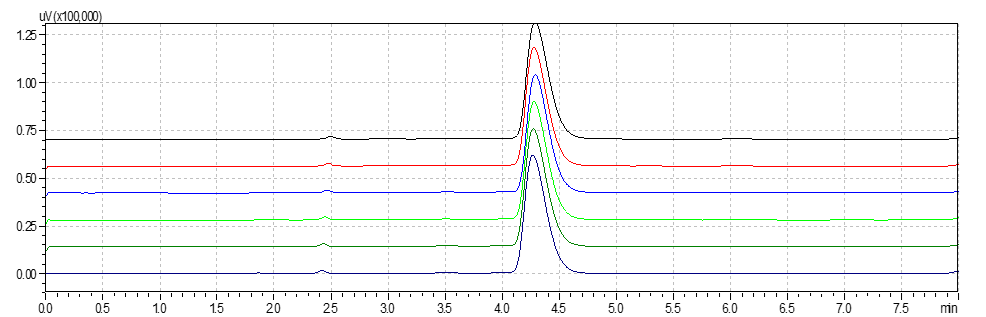

Supplement: Supplementary Figure S2 — The HPLC chromatograms of celecoxib in five consecutive association-dissociation cycles of ligand fishing assay eluent by SMGO-COX-2. [file Image_2.PNG]

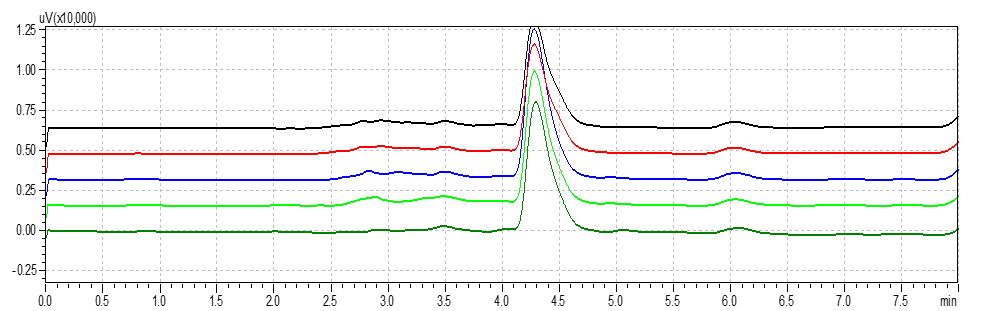

Supplement: Supplementary Figure S3 — The HPLC chromatograms of celecoxib in ligand fishing assay eluent by SMGO-COX-2 during five days of storage at 4°C. [file Image_3.PNG]
